# Changes in older employees’ willingness to utilise digital health promotion and prevention programmes during the SARS-CoV-2 pandemic

**DOI:** 10.1186/s12913-026-14398-1

**Published:** 2026-03-21

**Authors:** Jean-Baptist du Prel, Daniela Borchart

**Affiliations:** Department of Occupational Health Science, University of Wuppertal, Gaußstr. 20, D-42119 Wuppertal, NRW Germany

**Keywords:** Willingness to utilise, Digital workplace health promotion offers, Corona pandemic, Change, Older workers

## Abstract

**Background:**

For many employees, the SARS-CoV-2 pandemic has led to changes in working conditions, with increased use of digital technologies. Digital health promotion and prevention programmes can open up new opportunities for participation by companies and employees. Older employees are often slower to adopt to new technologies. We investigated whether the willingness of older employees to utilise such digital offers changed during the SARS-CoV-2 pandemic, taking socio-demographic and work-related organisational factors into account.

**Methods:**

Data from 2,267 randomly sampled older employees born in 1959 or 1965, who were in socially insured employment in Germany in the 3rd (2018) and 4th wave (2022/2023) of the prospective lidA (living at work) cohort study, were analysed. The change in the willingness to utilise online-based interventions, health apps and knowledge transfer platforms during the SARS-CoV-2 pandemic was analysed according to socio-demographic and work-related aspects using the McNemar’s test.

**Results:**

While around 80% of older employees favoured non-digital workplace health promotion and prevention measures in 2018, by 2022/2023 it was only 70%. In 2018, only 31.8% were willing to use online-based intervention services, by 2022/2023 it was 48.0%. 50.1% of older employees were willing to use health apps in 2022/2023, 12.2% more than in 2018. In contrast, the increase in the use of online platforms for knowledge transfer was only 4.5%. In terms of willingness to utilise online platforms for knowledge transfer and online-based intervention supplies, the proportion of men, older employees and those with a low level of education, who had initially expressed a willingness to participate and no longer do so, was high after the pandemic. In contrast, the willingness to use health apps increased for almost everyone during this period.

**Conclusions:**

Overall, the willingness to utilise digital health promotion and prevention programmes increased among older employees between 2018 and 2022/2023. The circumstances of the SARS-CoV-2 pandemic may have increased the acceptance of digital appliances. However, the opposite trend observed among certain socio-demographic groups regarding knowledge transfer platforms could also be due to the overuse of digital tools during this period.

**Supplementary Information:**

The online version contains supplementary material available at 10.1186/s12913-026-14398-1.

## Background

Digital technologies have become an integral part of most people’s everyday lives. They are often used in environments where target groups for health promotion and prevention measures are easily accessible [1]. The workplace is one such setting in which employees from different socio-demographic backgrounds can be accessed for workplace health promotion and prevention programmes.

Older employees are a special target group for workplace health promotion and prevention measures from an individual and social perspective: Firstly, the risk of illness increases with age [2]. Secondly, older employees of the “baby boomer generation” in Germany, as in most European countries, make up a significant proportion of the labour force and are therefore very important for maintaining social security systems [3]. With the demographic change, the ambition of employers to keep qualified older employees in the company for as long as possible is rising [4].

During the SARS-CoV-2 pandemic, the risk of a severe course of illness for coronavirus was higher among older employees than among younger employees [5]. A digital health promotion and prevention offer (DiHPO) was therefore of additional importance for older employees during the SARS-CoV-2 pandemic: it was an opportunity to take advantage of such programmes without the risk of infection. A positive effect of DiHPO on the health of older employees was already demonstrated before the pandemic [6].

However, older employees often show less preference for digital than conventional workplace health promotion and prevention programmes compared to younger employees [7]. In a pre-pandemic study, the preference of older employees in Germany was overwhelmingly in favour of non-digital workplace health promotion and prevention services [8]. This may be due to the fact that older employees often have less digital knowledge and skills compared to younger employees [9]. For the latter, the use of digital technologies often played a greater role in early phases of their education or professional biography than for older people. In contrast, the “baby boomer” cohorts considered in this study are sometimes also referred to as “digital immigrants”, as they were only able to adapt to many technical innovations through digitalisation during adulthood, due to the time of development of these appliances [10]. Due to this late “digital socialization” and a less favourable attitude and confidence toward new technologies than younger employees, older employees are also less motivated to use the latest information and communication technologies compared to younger employees [11].

Previous studies have also indicated socio-demographic differences among older adults concerning the use of digital technologies, in favour of the younger, the better educated and the financially better off, compared to the older, less educated and less financially well off [10]. In addition, the use of digital tools during work and the resulting training opportunities were relevant. Furthermore, occupational sector specific demands can have an influence how age interacts with technology adaption, making the relationship between age and technology adaption more nuanced [12]. For older employees who prefer non-digital services, being overwhelmed by digital technologies can also play a role [13]. In addition to digital competence and preference, the implementation of digital health promotion and prevention services in a home office setting also requires the appropriate technical and spatial equipment, which is not available to all employees [7].

The SARS-CoV-2 pandemic has led to the increased use of digital technologies in many areas of healthcare and treatment [14]. During the SARS-CoV-2 pandemic, significantly more employees than before were working from home, due to the risk of infection [13]. Around a third of older employees who worked from home during the pandemic showed an improvement in digital skills [9]. However, it was found that different social groups benefited differently in their digital skills from the pandemic-related changes. During the SARS-CoV-2 pandemic, not everyone had equal opportunities to work from home; predominantly intellectually active, higher-skilled and better-paid employees had easier access to remote work compared to low-skilled and predominantly physically active workers [15]. An improvement in digital skills was observed primarily among women, younger employees and those from the tertiary sector [9]. Even before the pandemic, female and the younger baby boomers showed a greater preference for digital tools [8]. Accordingly, the SARS-CoV-2 pandemic may have contributed to a pre-existing “digital divide” - the gap in equity that arises when digital health technologies disproportionally benefit privileged groups, while limited access, engagement, or culturally relevant design exacerbates health inequities - being aggravated [16].

In addition to reducing the risk of infection, the utilisation of DiHPO can also offer numerous advantages even after the SARS-CoV-2 pandemic. These include the greater flexibility of DiHPO in terms of location and time compared to face-to-face services [13], the possibility of personalisation, as well as cost savings (e.g., through the reutilisation of health promotion and prevention materials in networks) and time savings due to short distances and absence of waiting lists [17]. This can also be associated with an offer for target groups that are difficult to reach, such as night or shift workers [18], and for small and medium-sized enterprises [7]. In addition, there is a greater variety of services if DiHPO are provided alongside face-to-face programmes [17]. This is offset by the need for digital skills, and technical as well as spatial equipment for using digital services in home offices, a lack of social control over participation in DiHPO compared to face-to-face health promotion and prevention services and possible restrictions regarding data protection in DiHPO [7]. Inadequate reliability and quality of certain DiHPO can entail further risks [4]. Another difficulty is data protection, when health trackers are used to collect personal data that may also be processed for other purposes for example [19]. Another disadvantage of DiHPO compared to conventional measures of work health promotion and prevention are the limited possibilities of social interaction [20]. Table 1 summarises the advantages and disadvantages of DiHPO.

In this context, we hypothesize that the preference for workplace health promotion and prevention services among older employees has shifted in favour of DiHPO in the wake of the changed framework conditions during the SARS-CoV-2 pandemic. Furthermore we hypothesize that the differences in the preference for services between groups of different age, gender, education and professional requirements have changed during the pandemic. The latter could then have contributed to a widening or equalising of a pre-existing digital divide. With these preliminary considerations in mind, it was also of interest to investigate the correlation between company size and working hours and the development of DiHPO utilisation preferences during the SARS-CoV-2 pandemic.

**Table 1:** Advantages and disadvantages of digital health promotion and prevention programmes

Advantages DiHPO	Disadvantages DiHPO
Flexibility in terms of location and time [13]	Need for digital expertise [7]
Cost & time savings [17]	Requirement for technical equipment [7]
Opportunity of a health promotion offer for small & medium-sized companies [7]	Space requirement in the home office
Offer for hard-to-reach target groups [18]	Not every DiHPO is suitable for every target group [18]
Highly attractive, especially for female and younger employees [8]	Possibility of older employees in particular being overtaxed [7]
Anonymous participation possible	Limited opportunities for social interaction [20]
Increased variety as an additional service [17]	Lack of social control of participation
Better access to information	“Digital divide”, not all social groups have the same access opportunities [16]
Possibility of personalisation or individualisation [17]	Difficulties of ensuring data protection [17]
Reducing the risk (or fear) of infection	Poor reliability and quality of many DiHPO [4, 19]

Abr.: DiHPO = Digital Health Promotion and Prevention Offers

## Methods

The lidA (living at work) study is a Germany-wide, representative, prospective cohort study on work, health and work participation among older employees born in 1959 and 1965, who are subject to social insurance contributions. The sample for the first wave of the study was drawn on 31 December 2009 by a two-stage random sampling process from the ‘Integrated Employment Biographies’ (IEB) of the Federal Employment Agency. First, 222 sampling points were randomly selected from 206 of the 12,227 German communities. Then individuals were randomly drawn within the chosen regions. A detailed description of the sampling procedure can be found in the lidA cohort profile [3]. The IEB includes all socially insured employees in Germany. This exploratory data analysis included data from the third and fourth study waves 2018 and 2022/2023. Participants born in 1965 or 1959 were either in the age of 52/53 or 58/59 in 2018 and 56/58 or 62/64 in 2022/23 (the interview period in the fourth study wave was over 10 months due to the SARS-CoV-2 pandemic). In both study waves a separate module on health promotion and prevention was implemented into the survey instrument. An English translation of the questions of this module used in this investigation can be found in Supplement 1. Participants were consulted by computer assisted personal interviews (CAPI) at home in study wave 3 and 4 or alternatively by telephone (CAPI by Phone) in study wave 4 to reduce the risk of infection during the SARS-CoV-2 pandemic [21]. Cross-sectional results of the older employees’ willingness to utilise digital health promotion and prevention offers in study wave 3 was presented at the annual meeting of the German Society of Medical Sociology 2019 [8]. First results of the longitudinal analysis of this topic were presented at the annual meeting of the German Society of Epidemiology 2023 [22].

The 2,267 older employees who were in socially insured employment for at least one hour per week during these periods - i.e. once before and once during the SARS-CoV-2 pandemic - were asked about their willingness to use digital health promotion and prevention measures. A comparison of the lidA study sample with the population of all socially insured employees in 2018 and 2022/2023, showed that the lidA sample is highly representative of the data from the IEB [21, 23]. Furthermore, a drop-out analysis showed almost no selectivity with regard to 16 socio-demographic characteristics in the 4th study wave compared to the 3rd study wave [21].

The following variables were included in the analysis:

### Willingness to utilise DiHPO

The change in willingness to use DiHPO was surveyed using three different types of digital appliances in study waves 3 and 4 (Supplement 1):

When asked whether respondents use online-supported interventions (e.g., mindfulness training), health apps (e.g., on nutrition tips) and online platforms for knowledge transfer (online services that provide access to evidence-based information, tools and best practices to support learning and exchange) in the area of health promotion and prevention, the response options in each case were “yes, I already do this”, “yes, I would be willing to do so” and “no”. As part of this analysis, the categories “yes, I already do this” and “yes, I would be willing to do so” were combined into one category “I already do this or I would be willing to do so”, as both response categories reflect the basic willingness to utilise the corresponding digital services.

### Socio-demographic factors

The willingness to use different DiHPO was stratified according to various socio-demographic factors: differences in the willingness to utilise DiHPO were analysed, stratified by birth year (1959, 1965), gender (male or female), level of education and job qualifications. The level of education was recorded using a score that summarises both school education and vocational training [24]. This was then categorised into three educational strata. The requirement level was determined according to kldb2010 – the official occupational classification system used in Germany, developed by the Federal Statistical Office and the Federal employment Agency – based on self-reported activity currently performed. According to this categorisation, no vocational training is generally required for a semi-skilled job (“semi-skilled/unskilled”), two to three years of vocational training for a specialist job (“specialist”), a master craftsman or technician qualification for a complex specialist job (“complex”) and a university education is required for a highly complex expert job (“highly complex”) [25].

### Organisational factors

Working hours were recorded separately in the cross-section for waves 3 and 4 using the categories of professional activity without shift or night work (“No shift/night work”), in shift work (“Shift work”), in night work (“Night work”) and in the combination of shift and night work (“Shift & night work”). Only those who were in the same working time category at both survey dates were included in the longitudinal analysis. The company size was recorded based on the number of employees in the companies in which the respondents worked and classified according to micro (up to 9 employees), small companies (10 to 49 employees), medium-sized companies (50 to 249 employees) and large companies (at least 250 employees) up to and including those with over 1,000 employees. The number of employees in the companies in which the study participants worked was recorded for the first time in study wave 4.

### Statistics

The data was analysed using complete case analysis. Differences in the willingness to use DiHPO over time were visualised using Sankey diagrams and frequency distributions with 95% confidence intervals. The differences in the willingness to use DiHPO in the various socio-demographic and work-related organisational groups were statistically analysed cross-sectionally using the chi-square test and longitudinally using the McNemar’s test. The significance level in this explorative data analysis was 0.05. The analyses were conducted with IBM SPSS Statistics (Version 28.0; IBM Corp., 2021).

## Results

**Fig. 1:**
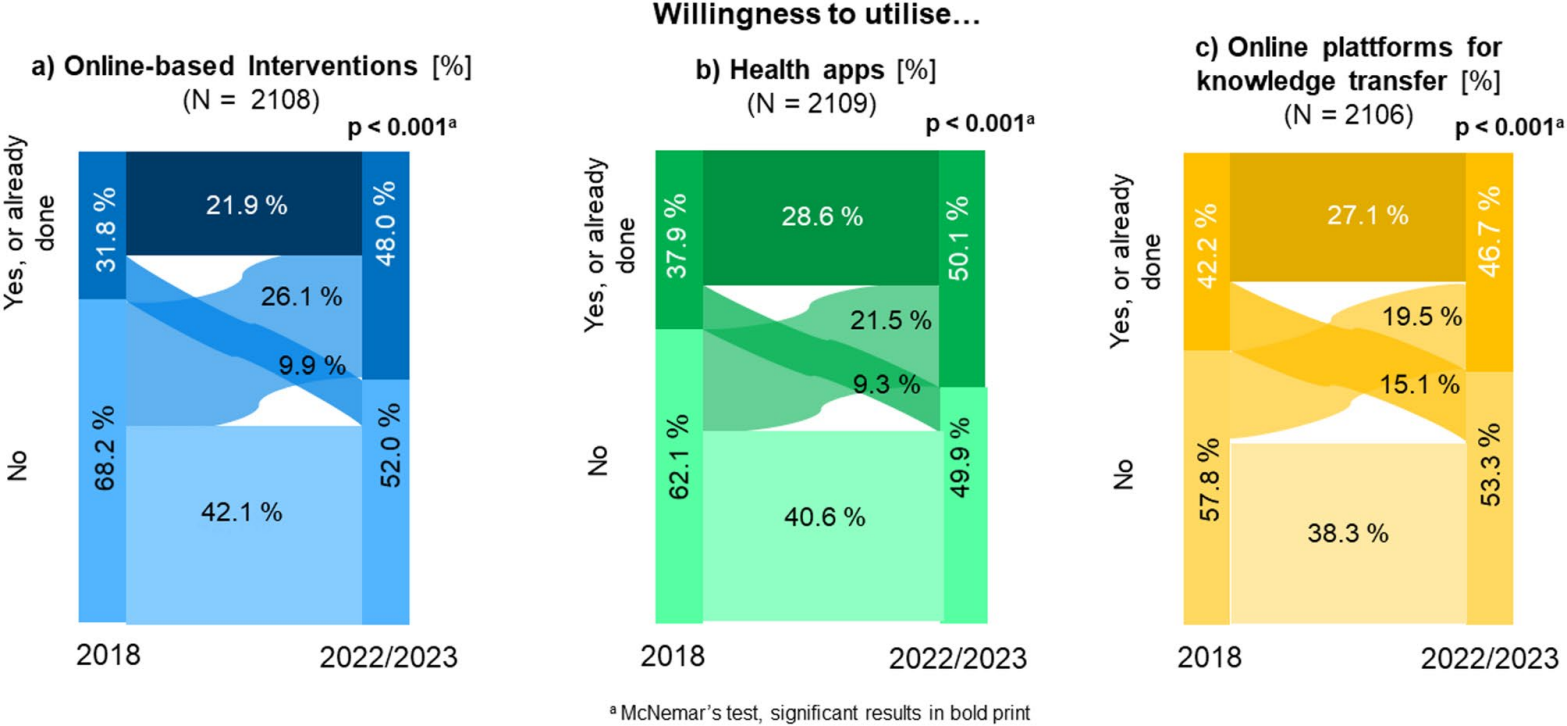
Change in the willingness of older employees to utilise DiHPO

The characteristics of the sample are given in Table 2. Figure 1a-c illustrates the changes in the willingness of the older employees to utilise the three different types of DiHPO between 2018 and 2022/2023. There were significant changes in the preference and willingness to use DiHPO over the two study waves. While around 80% preferred a non-digital health promotion and prevention offers at work in 2018, 30% preferred a DiHPO in 2022/23. There were changes in both directions over time in the willingness to use all types of DiHPO analysed (Fig. 1a-c). However, the proportions of switchers in the different directions were distributed differently depending on the type of DiHPO.

**Table 2:** Sample characteristics. Respondents of the lidA survey employed in the 3rd (2018) and 4th study wave (2022/2023). *N* = 2267

Characteristics	*n*	Percentage [%]	95%-CI [%]^c^
**Birth year**			
1959	884	39.0	37.0-41.1
1965	1380	61.0	58.9–63.0
**Sex**			
Male	1023	45.1	43.0-46.9
Female	1244	54.9	53.1–56.9
**Education**			
Low	412	18.3	16.8–19.8
Middle	1290	57.3	55.2–59.2
High	551	24.5	22.7–26.2
**Occupational requirement level** ^**a**^			
Semi-skilled/unskilled	127	5.7	4.7–6.8
Specialised	1208	54.3	52.1–56.6
Complex	423	19.0	17.3–20.7
Highly complex	467	21.0	19.3–22.5
**Working time** ^**b**^			
No shift work or night shift	1638	87.4	85.8–89.9
Shift work	117	6.2	5.1–7.4
Night shift	42	2.2	1.6-3.0
Shift work and night shift	78	4.2	3.4-5.0
**Company size** ^**a**^ (NoE)			
1 < 50	595	27.8	25.9–29.5
50 < 250	352	16.4	15.0-18.1
250 < 1000	377	17.6	15.9–19.2
≥ 1000	820	38.2	36.1–40.3

^**a**^ at study wave 4; ^b^only those employees who did not change their working time between study wave 3 and 4; ^c^ 95%-confidence interval for the true percentage in the target population; Abr.: NoE = Number of employees

### Online-based interventions

While only 31,8% older employees born in 1965 and 1959 were willing to use online-based interventions in 2018, 48,0% were willing to do so in 2022/2023. This change was due to the larger proportion of switchers in the direction of willingness to use online-based interventions (26.1%) compared to switchers in the opposite direction (9.9%) (Fig. 1a). The stratified analysis by gender, age, level of education, occupational requirement level and company size showed a significant increase in willingness to use online-based interventions between the two waves for all strata (Table 3). This increase was particularly pronounced for women, the younger baby boomers and those from large companies with at least 1,000 employees, i.e. groups in which the overall approval of utilisation was higher from the start. With regard to working hours, a significant increase in willingness to use the programme was only found among employees without shift or night shifts and among shift workers.

**Table 3:** Willingness of older employees to utilise DiHPO in the form of online-based intervention appliances 2018 and 2022/2023

Factors	Willingness to utilise online-based intervention supplies						
	Study wave 3 (2018)			Study wave 4 (2022/2023)			Trend (wave3 to 4) *p*-value (*n*)^c^
	Percentage [%]	95%-CI	*p*-value (*n*)^a^	Percentage [%]	95%-CI	*p*-value (*n*)^b^	
**Sex**			**0.018**			**< 0.001**	
Male	29.1	26.4–32.0	(978)	40.4	37.4–43.6	(947)	**< 0.001** (937)
Female	33.9	31.3–36.6	(1201)	54.0	51.2–56.9	(1186)	**< 0.001** (1171)
**Birth year**			0.679			**0.041**	
1959	31.2	28.2–34.4	(842)	45.2	41.8–48.6	(814)	**< 0.001** (800)
1965	32.1	29.6–34.6	(1334)	49.8	47.1–52.5	(1316)	**< 0.001** (1305)
**Education**			**0.006**			0.086	
Low	25.8	21.6–30.2	(396)	44.1	39.2–49.1	(390)	**< 0.001** (382)
Middle	31.9	29.4–34.5	(1241)	47.6	44.8–50.4	(1214)	**< 0.001** (1200)
High	35.6	31.6–39.8	(528)	51.5	47.1–55.8	(515)	**< 0.001** (512)
**Occupational requirement level** ^**d**^			0.400			0.114	
Semi-skilled/ unskilled	28.5	21.0-36.9	(123)	41.7	33.1–50.6	(120)	**0.032** (120)
Specialised	31.7	29.1–34.4	(1178)	46.7	43.9–49.6	(1158)	**< 0.001** (1144)
Complex	35.1	30.5–39.9	(399)	52.2	47.2–57.1	(389)	**< 0.001** (384)
Highly complex	30.5	26.4–35.0	(442)	49.8	45.1–54.5	(430)	**< 0.001** (426)
**Working time** ^**e**^			0.359			0.124	
No shift work or night shift	32.4	30.2–34.6	(1677)	49.0	46.6–51.3	(1716)	**< 0.001** (1573)
Shift work	27.6	21.9–33.8	(207)	42.5	35.9–49.3	(203)	**0.002** (117)
Night shift	35.2	25.9–45.3	(90)	45.2	35.3–55.3	(93)	0.549 (35)
Shift work and night shift	28.6	21.7–36.2	(144)	37.9	29.5–47.0	(116)	0.281 (78)
**Company size** (NoE)^**d**^			0.631			**0.008**	
1 < 50	31.8	28.1–35.8	(556)	45.7	41.6–49.9	(551)	**0.015** (538)
50 < 250	29.6	24.9–34.5	(345)	43.2	38.1–48.4	(352)	**0.032** (344)
250 < 1000	31.0	26.5–35.8	(371)	46.5	41.5–51.6	(374)	**0.001** (366)
≥ 1000	33.3	30.1–36.6	(808)	52.6	49.2–56.1	(815)	**< 0.001** (798)

^**a**^Analysis of **c**ross-sectional group differences 2018 using chi-square-test ^**b**^ Analysis of cross-sectional group differences 2022/2023 using chi-square-test ^c^Analysis of longitudinal group differences between 2018 and 2022/2023 using McNemar’s test; significant results (*p* < 0,05) in bold print; ^d^measured in study wave 4; ^e^measured cross-sectionally separately in study waves 3 and 4 and longitudinally with matching working time conditions in wave 3 and 4; Abr.: DiHPO = digital health promotion and prevention offers; NoE = Number of employees

### Health apps

The frequency of willingness to use health apps increased by 12.1% to 50.1% during the pandemic (Fig. 1b). Here too, the proportion of switchers in the direction of willingness to use (21.5%) outweighed those in the other direction (9.1%). There was also a significant increase in the willingness to use in almost all strata of the socio-demographic groups between the waves, with the exception of semi-skilled and unskilled workers and those in night or night and shift work (Table 4).

**Table 4:** Willingness of older employees to utilise DiHPO in the form of health apps 2018 and 2022/2023

Factors	Willingness to utilise health apps						
	Study wave 3 (2018)			Study wave 4 (2022/2023)			Trend (wave3 to 4) *p*-value (*n*)^c^
	Percentage [%]	95%-CI	*p*-value (*n*)^a^	Percentage [%]	95%-CI	*p*-value (*n*)^b^	
**Sex**			**< 0.001**			**< 0.001**	
Male	33.5	30.6–36.5	(978)	45.0	41.9–48.2	(948)	**< 0.001** (938)
Female	41.2	38.4–44.0	(1202)	54.4	51.6–57.3	(1185)	**< 0.001** (1171)
**Birth year**			**0.005**			**< 0.001**	
1959	34.2	31.0-37.4	(843)	45.2	41.8–48.6	(814)	**< 0.001** (801)
1965	40.1	37.5–42.8	(1334)	53.4	50.7–56.1	(1316)	**< 0.001** (1305)
**Education**			**0.025**			0.251	
Low	32.0	27.5–36.7	(397)	46.5	41.6–51.5	(389)	**< 0.001** (382)
Middle	39.6	36.9–42.3	(1241)	51.4	48.5–54.2	(1215)	**< 0.001** (1201)
High	38.1	34.0-42.3	(528)	50.5	46.2–54.8	(515)	**< 0.001** (512)
**Occupational requirement level** ^**d**^			0.226			0.494	
Semi-skilled/ unskilled	39.8	31.5–48.6	(123)	45.8	37.1–54.8	(120)	**0.382** (120)
Specialised	36.2	33.5–38.9	(1178)	49.6	46.7–52.5	(1157)	**< 0.001** (1143)
Complex	38.3	33.7–43.2	(399)	50.9	45.9–55.8	(389)	**< 0.001** (384)
Highly complex	41.6	37.1–46.3	(442)	52.9	48.2–57.6	(431)	**< 0.001** (427)
**Working time** ^**e**^			**0.040**			0.828	
No shift work or night shift	37.4	35.1–39.7	(1727)	50.9	48.5–53.2	(1716)	**< 0.001** (1574)
Shift work	39.7	33.3–46.4	(214)	50.2	43.4–57.1	(203)	**0.002** (117)
Night shift	38.5	29.0-48.7	(91)	48.4	38.4–58.5	(93)	0.065 (35)
Shift work and night shift	38.8	31.2–46.8	(147)	44.0	35.2–53.1	(116)	0.248 (78)
**Company size** (NoE)^**d**^			**0.024**			**0.013**	
1 < 50	33.5	29.6–37.5	(556)	45.6	41.5–49.8	(550)	**< 0.001** (546)
50 < 250	36.7	31.8–41.9	(346)	48.6	43.4–53.8	(352)	**< 0.001** (346)
250 < 1000	39.9	35.0-44.9	(371)	49.5	44.4–54.5	(374)	**0.002** (370)
≥ 1000	41.3	38.0-44.8	(808)	54.4	51.0-57.8	(816)	**< 0.001** (808)

^**a**^Analysis of cross-sectional group differences 2018 using chi-square-test ^**b**^ Analysis of cross-sectional group differences 2022/2023 using chi-square-test ^c^Analysis of longitudinal group differences between 2018 and 2022/2023 using McNemar’s test; significant results (*p* < 0,05) in bold print; ^d^measured in study wave 4; ^e^measured cross-sectionally separately in study waves 3 and 4 and longitudinally with matching working time conditions in wave 3 and 4; Abr.: DiHPO = digital health promotion and prevention offers; NoE = Number of employees

While there were mostly only gradual differences in the willingness to utilise health apps, the increase was particularly pronounced among the less educated. An educational gradient, which was observed in the third study wave, no longer existed in the fourth study wave. Regarding different working time patterns, a significant difference in willingness to use health apps that was still observable in the third wave of the study, also no longer existed in the fourth wave.

### Online platforms for knowledge transfer

The slower growth in the utilisation of online platforms for knowledge transfer (less than 5%), on the other hand, was due to the fact that the difference of the proportion of switchers in both directions was not particularly high (Fig. 1c). The subgroup analysis of the socio-demographic group differences showed that the difference between the study waves in the proportion of those who were willing to use online platforms for knowledge transfer was small, especially among men and those with a low level of education (Table 5). The most significant increases were again among female employees and those from large companies with more than 1,000 employees, who also showed the highest proportion of willingness to use this medium in their group.

**Table 5:** Willingness of older employees to utilise DiHPO in the form of online platforms for knowledge transfer 2018 and 2022/2023

Factors	Willingness to utilise online platforms for knowledge transfer						
	Study wave 3 (2018)			Study wave 4 (2022/2023)			Trend (wave3 to 4) *p*-value (*n*)^c^
	Percentage [%]	95%-CI	*p*-value (*n*)^a^	Percentage [%]	95%-CI	*p*-value (*n*)^b^	
**Sex**			**0.030**			**< 0.001**	
Male	40.1	37.0-43.2	(976)	42.7	39.5–45.8	(947)	0.144 (935)
Female	44.7	41.9–47.2	(1202)	50.0	47.1–52.8	(1185)	**0.001** (1171)
**Birth year**			**0.004**			**0.018**	
1959	38.8	35.5–42.1	(843)	43.5	40.1–46.9	(814)	**0.009** (801)
1965	45.0	42.4–47.7	(1332)	48.7	46.0-51.4	(1315)	**0.022** (1302)
**Education**			**< 0.001**			**< 0.001**	
Low	32.0	27.5–36.7	(397)	34.7	30.1–39.5	(389)	0.400 (382)
Middle	43.1	40.4–45.9	(1240)	47.8	45.0-50.6	(1214)	**0.002** (1199)
High	49.3	45.1–53.6	(527)	53.2	48.9–57.5	(515)	0.168 (511)
**Occupational requirement level** ^**d**^			**< 0.001**			**< 0.001**	
Semi-skilled/ unskilled	26.0	18.9–34.3	(123)	35.0	26.9–43.8	(120)	0.382 (120)
Specialised	40.1	37.3–42.9	(1178)	43.8	40.9–46.6	(1156)	**0.018** (1142)
Complex	50.5	45.6–55.4	(398)	51.7	46.7–56.6	(389)	0.661 (383)
Highly complex	47.4	42.8–52.1	(441)	52.7	47.9–57.4	(431)	0.094 (426)
**Working time** ^**e**^			0.193			0.522	
No shift work or night shift	43.8	41.4–46.8	(1725)	47.5	45.1–49.8	(1715)	**0.003** (1571)
Shift work	37.4	31.1–44.0	(214)	43.3	36.7–50.2	(203)	0.229 (117)
Night shift	37.4	27.9–47.6	(91)	46.2	36.3–56.4	(93)	1.000 (35)
Shift work and night shift	40.1	32.5–48.2	(147)	42.2	33.5–51.3	(116)	0.345 (78)
**Company size** (NoE)^**d**^			**0.010**			**< 0.001**	
1 < 50	37.6	33.6–41.7	(556)	41.0	36.9–45.1	(549)	0.150 (545)
50 < 250	40.5	35.4–45.7	(346)	47.2	42.0-52.4	(352)	0.062 (346)
250 < 1000	45.6	40.5–50.6	(371)	43.6	38.6–48.6	(374)	0.543 (370)
≥ 1000	45.9	42.5–49.4	(806)	52.6	49.1–56.0	(816)	**< 0.001** (806)

^**a**^Analysis of **c**ross-sectional group differences 2018 using chi-square-test ^**b**^ Analysis of cross-sectional group differences 2022/2023 using chi-square-test ^c^Analysis of longitudinal group differences between 2018 and 2022/2023 using McNemar’s test; significant results (*p* < 0,05) in bold print; ^d^measured in study wave 4; ^e^measured cross-sectionally separately in study waves 3 and 4 and longitudinally with matching working time conditions in wave 3 and 4; Abr.: DiHPO = digital health promotion and prevention offers; NoE = Number of employees

## Discussion

The willingness and preference to use DiHPOs among older employees subject to social insurance increased overall between 2018 and 2022/2023. Many employees worked from home during the SARS-CoV-2 pandemic, which fell in this period, and companies often switched partially or completely to DiHPO [7]. The special circumstances of the SARS-CoV-2 pandemic have led to an improvement in digital skills of older employees [9]. This may have led to an increase in the acceptance of DiHPO, too. The increase in willingness to use online-supported interventions and health apps was particularly pronounced, but less so for online platforms for knowledge transfer. However, the willingness to use the latter was already at a comparatively high level before the pandemic.

The relatively large proportion of users who were willing to use them before the pandemic and were no longer willing to use them at the second observation point can also explain the slight increase. The countertrend in the willingness to use digital tools that was observed more frequently in these groups and in certain socio-demographic groups (male and less educated older employees), could be due to overuse of digital tools during the SARS-CoV-2 pandemic with a consecutive fatigue effect. An online fatigue effect and a consequent decline in usage of online platforms for knowledge transfer have also been described for students in the SARS-CoV-2 pandemic [26, 27].

It is difficult to see that the increase in willingness to use digital tools often occurred in groups that had already signalled a greater willingness to use digital technologies before the pandemic, such as older female employees, the younger among the older employees and employees from large companies. This could exacerbate a pre-existing digital divide. After all, it is not just the (additional) offer of digital health promotion and prevention measures that is sufficient, but also the acceptance of the offer that determines their real benefit. In theory, as described above, a DiHPO appears to be of great advantage for small and medium-sized companies that may not be able to afford their own company health promotion programme [7]. However, this study showed that older employees from small and medium-sized companies in particular, are less willing to use DiHPO than those from large companies, and that the increase in willingness to utilise such offers during the SARS-CoV-2 pandemic was also lower there. The same applied to DiHPO for target groups that are difficult to reach [18], such as older male employees. Here, too, the willingness to use DiHPO and its growth was lower than with female employees. The disadvantages of DiHPO already mentioned, such as the lack of opportunity to socialise with others [20], could play a role in the groups with a lower willingness to use DiHPO or a lower increase of their willingness to do so during the SARS-CoV-2 pandemic. This pandemic in particular, has led to isolation for many people, which more frequently affected the target groups we identified as also being difficult to reach for DiHPO, such as men, older employees and the less educated [28]. Further studies need to examine the barriers to DiHPO utilisation in hard-to-reach groups in more detail in order to overcome these obstacles.

In this context, however, the fact that in contrast to the other DiHPOs, the increase in willingness to use health apps was more pronounced among the less educated can be seen as favourable for reducing the digital divide. While a social gradient in the willingness to use apps in favour of the less educated could still be observed before the pandemic [8], there was no longer any significant difference between the education groups afterwards. The SARS-CoV-2 pandemic has evidently helped to largely eliminate educational differences in the willingness to use health apps. Health apps therefore now represent an opportunity to better reach less educated older employees, a group that is difficult to reach with analogue health promotion and prevention services [29].

The results on an overall increasing willingness to use DiHPO among older employees are promising from the point of view that a current systematic review, including 17 randomised controlled intervention studies, was able to demonstrate the health-related effectiveness of various DiHPO measures for exercise, stress management and weight reduction [30]. This also showed that health literacy of the participants is of great importance for the success of the measures. The motivation of the participants also proved to be a decisive factor for lasting success.

### Practical implications

Improving health literacy, e.g., in less educated older employees and those with G1 migration background, and increasing motivation, e.g., through DiHPO specifically tailored to certain target groups (e.g., male older employees), could be a way of increasing the willingness of the hard-to-reach target groups identified in our study to take part.

### Strength and limitations

A strength of the lidA study is its representativeness of socially insured employees born in 1959 or 1965 working in Germany [3]. Furthermore, the study provides longitudinal data for a high number of study subjects across four study waves. Another strength is the great variety of study characteristics included in the lidA study, enabling us to investigate individual, as well as work-related organisational factors with regard to the older employees’ willingness to utilise three different types of digital health promotion and prevention appliances.

Beside these merits, our investigation has several limitations. Only two age cohorts (1959, 1965) of older employees subject to social insurance contributions in Germany were included in this study. In the fourth wave, due to the corona pandemic, the CAPI-by-phone survey was used as an alternative to the CAPI survey type [21]. The choice between both survey types was voluntary. A separate analysis (not shown here), showed that respondents who had a higher preference for digital technologies were more likely to opt for the CAPI-by-phone variant. However, a comparison of the results of the two survey types showed only slight selectivity [21]. Moreover, we used two different questions on the use of DiHPO tools. First, we asked the participants about their general willingness to utilize three different DiHPO tools. We assumed that people who are generally willing to utilize DiHPO tools would be also willing to use them at the working place. Especially in the situation of the SARS-CoV-2 pandemic, where many employees worked at home, a distinction between the willingness to use DiHPO tools at work or at home seemed not to be suitable. Nevertheless, there might be reasons why people are willing to utilize it at home and not at work in the non-pandemic situation. Then we asked whether the participants would prefer conventional or digital DiHPO tools at the working place. As we asked both questions before and in the pandemic, it is likely that these changes in willingness to utilize DiHPO tools are due to the situation during the pandemic.

Another limitation is that we only surveyed the size of the company in the fourth wave of the study. The results on the change in willingness to use digital health promotion and prevention programmes depending on the company size, are therefore only accurate if the employees did not switch between different sized companies between 2018 and 2022/2023. However, according to our data, only 13.7% of the older employees included in this study had changed their employer between the 3rd and the 4th wave of the lidA-study. Even for the unlikely case if all of them had changed to an employer in a different sized company it would not have a great impact on our results.

Last but not least our primary objective was to assess respondents’ overall motivational readiness to utilize digital technologies in the area of health promotion and prevention rather than to differentiate between behavioural enactment and behavioural intention. Combining the categories “yes, I already do” and “I would be willing to do so” to a single indicator of positive behavioural motivation is consistent with the interpretation of willingness and current behaviour as reflecting a favourable motivational orientation toward the target behaviour. Our focus was not on the distinction between intention and behaviour where the intention-behaviour gap could be a relevant aspect.

## Conclusions

Future surveys will show whether the trend towards a greater willingness to use DiHPO will continue after the SARS-CoV-2 pandemic. Such a development would be favourable with regard to the opportunities offered by DiHPO for small and medium-sized companies, for target groups that are difficult to reach (e.g., shift workers), when working from home or as an additional offer to conventional health promotion and prevention services. However, DiHPO would then have to be accompanied by additional educational work and advertising of these digital workplace health promotion and prevention measures, especially in hard-to-reach target groups, so that these digital alternative programmes will actually be utilised. Digital skills also need to be expanded, especially among older employees and particularly among certain groups who are less willing to use them, in order to ensure access to these services and reduce the digital divide in terms of usage [31]. In further studies, the acceptance barriers of target groups that are difficult for DiHPO to reach should be investigated in more detail. DiHPO have the potential to increase the cost-effectiveness of health interventions [1]. However, data protection should be taken into account when individualising DiHPO.

## Supplementary Information

Below is the link to the electronic supplementary material.

Supplementary Material 1

## Data Availability

The datasets for the first two waves of the lidA study are available as a scientific use file at the research data centre of the German Federal Employment Agency at the Institute of Employment Research. Further information can be found here: https://fdz.iab.de/en/our-data-products/archived-data/lida/ [32]. The scientific use file for study wave 3 is still in preparation and will be available for researchers at the Research Data Centre of the German Statutory Pension Insurance in the near future. Additional information regarding the study, as well as data documentation (data reports and methods reports) are also available [3, 21, 22, 33, 34].

## Abbreviations

DiHPO: Digital Health Promotion and Prevention Offers
lidA: living at work
