# Supplementary material for: Changes in older employees’ willingness to utilise digital health promotion and prevention programmes during the SARS-CoV-2 pandemic
Source: BMC Health Serv Res. 2026 Mar 21;26:490. doi: 10.1186/s12913-026-14398-1 (PMC13063842; doi:10.1186/s12913-026-14398-1)
Supplement: Supplementary file 1 — Supplementary Material 1 [file 12913_2026_14398_MOESM1_ESM.docx]

**Supplement 1** Questions from the health promotion and prevention module of the lidA survey in study waves 3 and 4

*Willingness for digital interventions*

There are an increasing number of digital health programmes on the health market that are designed to motivate people to exercise more, eat healthy food for example, or to strengthen their personal health skills.

Which of the digitally supported health promotion programmes listed below would you be willing to take part in?

A: Online-supported interventions such as back exercises or mindfulness training

B: Health apps, e.g. for measuring steps or nutrition tips

C: Online platforms for knowledge transfer

D: Other digitally supported measures

1: Yes, I already do this

2: Yes, I would be willing to do so

3: No

97: Refused response

98: Don't know

*Preference for digital & non-digital measure*

If you had to choose, which would you prefer in a company context?

A non-digital health promotion measure (e.g. back training in the workplace) or a digitally supported health promotion measure (e.g. online back training)?

1: A non-digitally supported measure

2: A digitally supported measure

3: I consider both to be of equal value and prefer neither

97: Refused response

98: Don't know
